# Non-pyrogenicity and biocompatibility of parylene-coated magnetic bead implants

**DOI:** 10.3389/fbioe.2024.1290453

**Published:** 2024-02-20

**Authors:** Cameron R. Taylor, Joshua K. Nott, Nilmini H. Ratnasena, Joel M. Cohen, Hugh M. Herr

**Affiliations:** ^1^ K. Lisa Yang Center for Bionics, Massachusetts Institute of Technology, Cambridge, MA, United States; ^2^ RQM+, Mansfield, MA, United States; ^3^ Gradient Corp, Boston, MA, United States

**Keywords:** human-machine interfaces, implantable technology, biocompatibility, muscle tracking, wearable technology, magnetomicrometry, bionics, prosthetic control

## Abstract

Clinical grade magnetic bead implants have important applications in interfacing with the human body, providing contactless mechanical attachment or wireless communication through human tissue. We recently developed a new strategy, magnetomicrometry, that uses magnetic bead implants as passive communication devices to wirelessly sense muscle tissue lengths. We manufactured clinical-grade magnetic bead implants and verified their biocompatibility via intramuscular implantation, cytotoxicity, sensitization, and intracutaneous irritation testing. In this work, we test the pyrogenicity of the magnetic bead implants via a lagomorph model, and we test the biocompatibility of the magnetic bead implants via a full chemical characterization and toxicological risk assessment. Further, we test the cleaning, sterilization, and dry time of the devices that are used to deploy these magnetic bead implants. We find that the magnetic bead implants are non-pyrogenic and biocompatible, with the insertion device determined to be safe to clean, sterilize, and dry in a healthcare setting. These results provide confidence for the safe use of these magnetic bead implants in humans.

## 1 Introduction

Implantable magnetic beads have the potential to provide new interfaces with the human body. For instance, in a new technique that we call magnetomicrometry (Taylor et al., 2022a), the distance between two magnetic beads implanted in muscle is tracked similarly to the techniques of fluoromicrometry (Camp et al., 2016) and sonomicrometry (Griffiths, 1987), but magnetomicrometry can be performed in real-time without heavy equipment or percutaneous wires. However, for magnetomicrometry to be used in humans, the clinical viability of magnetic bead implants must first be verified. In previous work, we addressed the salient aspects of the clinical viability of implanting magnetic beads in muscle (Taylor et al., 2022b). Namely, we described a manufacturing process for medical-grade magnetic bead implants, in which we coat 3-mm-diameter neodymium spheres in 5 µm of gold and 21 µm of Parylene C and ultrasonically-clean, magnetize, package, and gamma-sterilize them, and we also described the manufacturing of implant insertion devices that are used to deploy the magnetic bead implants from their cartridge packaging. Using these implants, deployed via these insertion devices, we found no evidence of discomfort from the implants, and we confirmed that the implants are non-irritant, non-cytotoxic, non-allergenic, and non-irritating. However, before obtaining FDA approval to use these implants in a first-in-human trial, it was also necessary to perform implant pyrogenicity testing, chemical characterization, and toxicological risk assessment, and to test the cleaning, sterilization, and dry time of the implant insertion device. This work presents the methods and results of those additional tests. Noting that Iacovacci et al. (2021) showed that 3-mm-diameter 1-mm-thick neodymium disc magnets coated with 10 µm of Parylene C are non-pyrogenic and systemically non-toxic, in this investigation we hypothesize that the beads are non-pyrogenic under pyrogenicity testing and will be found to be biocompatible under toxicological risk assessment. Further, noting that we manufactured the insertion device using the same components as an already-clinically-available device with minimal changes, we also hypothesize that the implant insertion device can be safely cleaned, sterilized, and dried in a healthcare setting.

In this work, we verify the non-pyrogenicity of the magnetic bead implants and submit the implants to a full chemical characterization and toxicological risk assessment. Further, we validate the cleaning efficacy, steam sterilization, and dry time of the magnetic bead insertion device. All testing is performed under good laboratory practice. We believe these results to be valuable to further scientific progress in the use of magnetic bead implants for human-machine interfacing.

## 2 Materials and methods

### 2.1 Pyrogenicity testing

To perform further biocompatibility testing on the device under good laboratory practice (GLP) compliance (USFDA, Code of Federal Regulations, Title 21, Part 58–Good Laboratory Practice for Nonclinical Laboratory Studies), we submitted fully manufactured magnetic bead sets and insertion devices to WuXi AppTec for pyrogenicity testing. All magnetic beads used in the testing were deployed from the magnetic bead cartridges using the insertion device, and the testing was conducted in compliance with international standard ISO 10993-12:2012, Biological Evaluation of Medical Devices, Part 12: Sample Preparation and Reference Materials.

The pyrogenicity testing protocols were reviewed and approved by the WuXi AppTec IACUC prior to the initiation of testing. Eight rabbits were used for pyrogenicity testing in this work. Albino rabbits (*Oryctolagus cuniculus*, young adult, female) were obtained from Charles River Laboratories and maintained in the WuXi AppTec animal facility according to NIH and AAALAC guidelines on an *ad libitum* (except during the test period) water and certified commercial feed diet.

To test for the induction of a febrile response, magnetic beads were extracted at a ratio of 3 cm^2^/1 mL (surface area per volume) into each of 150.0 and 250.3 mL of 9.0 g/L normal saline (1592 and 2656 magnetic beads for an initial test and a continued test, respectively). These extractions were freshly prepared for corresponding test phases and were performed over 72 h at 50°C, with agitation during the extraction, then warmed to 37°C immediately before use.

In an initial test, 10 mL/kg of the extraction was slowly injected into the marginal ear vein in each of the first three rabbits. An automated temperature recorder measured a baseline rectal temperature no more than thirty minutes before the injection and the maximum temperature between one and 3 hours post-injection (from measurements taken at 1, 1.5, 2, 2.5, and 3 h post-injection). This same testing was then performed in a continued test on the five additional rabbits. A temperature increase was calculated for each animal by subtracting the baseline temperature from the maximum temperature and rounding all negative temperature differences up to zero. If no temperature increase greater than 0.5°C had been observed in any of the animals in the initial test, the magnetic beads would have met the requirements of the test immediately following the initial test. The measurement of a temperature increase greater than 0.5°C in at least one animal of the initial test was considered grounds for performing a continued test, and a continued test was performed. As directed in the pyrogenicity testing procedure, fewer than three temperature increases greater than 0.5°C and a summed temperature increase of less than 3.3°C among the total of eight animals used in the initial and continued test were considered to indicate that the magnetic bead implants did not elicit a pyrogenic response. For a flowchart of the pyrogenicity testing process, please see Supplementary Figure S1. This pyrogenicity testing complied with United States Pharmacopeia (USP) Pyrogen Test Procedure, Section 151, with sample-specific preparation and extraction modifications as needed.

### 2.2 Chemical characterization and toxicological risk assessment

To evaluate the toxicological potential of the magnetic bead implants as permanent implant devices, we submitted fully manufactured magnetic bead sets and insertion devices to Jordi Labs for chemical characterization. Gradient Corp then evaluated the chemical characterization results in a toxicological risk assessment. All magnetic beads used in the testing were deployed from the magnetic bead cartridges using the insertion device, and the testing was conducted in compliance with ISO 10993-12:2021, Biological Evaluation of Medical Devices, Part 12: Sample Preparation and Reference Materials. The chemical characterization was conducted in compliance with ISO 10993-18:2020, Biological evaluation of medical devices—Part 18: Chemical characterization of medical device materials within a risk management process.

For the performance of the chemical characterization, magnetic beads were extracted at a ratio of 3 cm^2^/1 mL (surface area per volume) into four 4.93 mL borosilicate vials each of purified water, ethanol, and hexane (12 sets of 48 magnetic beads–576 beads total). Three of the vials for each extraction solvent were used to perform analysis in triplicate, while one vial was dedicated to gravimetric analysis. These extractions were performed by exhaustive submersion, with agitation during the extraction. The first extraction was performed over 72 h at 50°C, while subsequent rounds would have been repeated over 24 h at 50°C until the gravimetric analysis showed that the mass of the non-volatile residue (NVR) was less than 10% the mass of the first extraction. However, all extractions met the exhaustive extraction criteria after the first round, with a measured total residue of less than 0.002 mg/device (less than the reporting limit of 0.1 mg), so the exhaustive extraction was stopped after one round. A control extraction was identically performed for each extraction solvent.

For the detection, identification, and quantitation of volatile and non-volatile organic compounds from the magnetic bead extractions, 0.2 mL of each of the extractions was analyzed by gas chromatography mass spectrometry (GCMS), and 0.2 mL of each of the water and ethanol extractions and 0.5 mL of each hexane extraction were analyzed by quadrupole time of flight liquid chromatography mass spectrometry with ultraviolet-visible and charged-aerosol detection (QTOF-LCMS-UV-CAD). The water extractions were also analyzed by head-space GCMS (HSGCMS) for the detection of volatile organic compounds. Elemental extractables from the water extractions were then quantified using inductively coupled plasma mass spectrometry (ICP-MS). For an overview diagram of the distribution of how these extractions were used in these tests, please refer to Supplementary Figure S2. An analytical evaluation threshold (AET) was calculated as directed in the ISO standard, using a dose-based threshold of 1.5 ug/day and a maximum number of medical devices of sixteen. A dilution factor of four was used for QTOF-LCMS-UV-CAD, giving an AET concentration of 0.228 μg/mL, and a dilution factor of five was used for GCMS, giving an AET concentration of 0.182 μg/mL. A multidetector approach was used to reduce the effects of relative response factor variation and to provide an uncertainty factor to adjust the AET. For more details on the methodologies of this chemical characterization process, please refer to Jordi et al., 2020.

For the assessment of the identified chemicals for toxicological risk, an allowable limit for each chemical was calculated according to ISO Standard 10993-17:2002 using a minimum expected body weight of 58 kg. An exposure level was then conservatively calculated for each chemical using the assumption that the devices would daily release the maximum amount of chemical measured across all three solvents following the 72-h exhaustive extraction and multiplying this by an expected maximum of sixteen devices per patient. The allowable limit was divided by this exposure level to give a margin of safety for each chemical. A margin of safety greater than 10 for compounds and greater than 1 for elements was considered to indicate low toxicological risk for each evaluated chemical for systemic toxicity, mutagenicity, carcinogenicity, and reproductive and developmental toxicity.

### 2.3 Efficacy of cleaning the magnetic bead insertion device

To test the efficacy of manually cleaning the magnetic bead insertion device for healthcare settings, we submitted three magnetic bead insertion devices to WuXi AppTec for cleaning efficacy testing.

We used the following instructions for use as the proposed cleaning process for the magnetic bead insertion devices: Disassemble the device into its four components, soak the components in a neutral-pH enzyme cleaner (Alconox Tergazyme, 10 g/L aqueous solution at room temperature) for 20 minutes, and manually clean each component with scrub brushes and straw cleaners. Then, rinse each component with tap water. Next, sonicate the device components in a neutral-pH detergent (Alconox Luminox, 30 mL/L aqeuous solution at room temperature) for 10 minutes, and rinse each component of the device with tap water for 1 minute, then repeat the sonication and rinsing steps once more. Finally, wipe the devices with clean disposable non-shedding wipes (Kimwipes) and transfer the disassembled devices immediately to autoclave pouches for sterilization.

**FIGURE 1:**
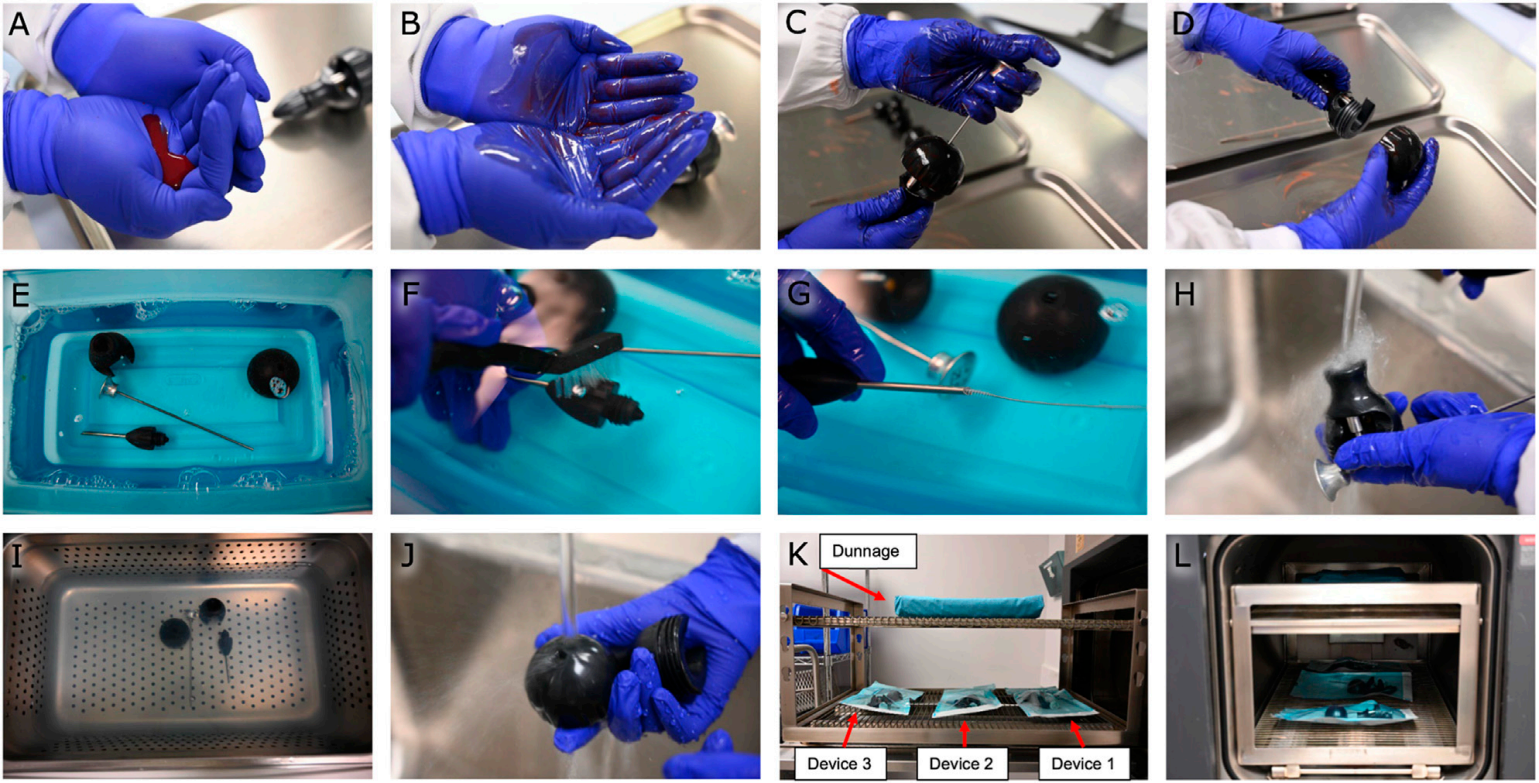
Cleaning efficacy study. **(A)** Preparation of artificial soil. **(B)** Spreading the artificial soil on gloved hands. **(C,D)** Handling and manipulating the device with soiled gloved hands. **(E)** Soaking the device in an enzyme cleaner. **(F,G)** Manually cleaning the device with scrub brushes and straw cleaners. **(H)** Rinsing the device with tap water. **(I)** Sonicating the device. **(J)** Rinsing the device with tap water. **(K)** Placing the devices on the autoclave rack with dunnage overhead. **(L)** Autoclaving the devices at 134°C.

A full simulated-use cycle (See Figure 1) was defined as soiling each device, cleaning the devices with a worst-case cleaning process, then running the devices through a full autoclave cycle. To soil the devices, 2 mL of prepared artificial soil (ATS2015 soil–Healthmark) was spread on gloved hands, and the soiled gloved hands were used to handle and manipulate the device to spread the soil across all surfaces. The devices were then left to air-dry for a minimum of 2 hours. The worst-case cleaning process was chosen by decreasing the time and the cleaner concentrations of each step of the above proposed cleaning process (10 minutes of soaking in a 5 g/L enzyme cleaner solution, five-minute sonications in 15 mL/L aqeuous solution detergent, and forty-five-second rinses), then allowing the devices to air-dry instead of wiping them down. Devices were autoclaved in a three-minute pre-vacuum cycle at 134°C, followed by a thirty-minute dry time.

For the testing of remaining soil on the devices, each disassembled device was hand-shaken in an extraction bag with 200 mL of water for 1 minute, then sonicated in the bag for 10 minutes, and hand-shaken for one additional minute. The sample extract was then tested for residual protein (via Micro BCA) and total organic carbon using quantitative colorimetric test methods.

One device served as a negative device control before any other testing occurred. The negative device control was cleaned according to the worst-case scenario (Figures 1E–J) and then tested for remaining soil to verify that the measured soil quantities were at or slightly above negative sample controls. All three devices then served as positive device controls. The positive device controls underwent six full simulated-use cycles (Figures 1A–L). They were then soiled once (Figures 1A–D) and extracted three times to verify that a correction factor was unnecessary to account for recovery efficiency. Positive sample controls were then used to ensure the validity of the soil test assays.

For the testing of cleaning efficacy when manually cleaning the devices, two manual cleaning efficacy cycles were then performed on all three devices by soiling the devices, then cleaning them following the worst-case cleaning process (Figures 1A–J). After each manual cleaning efficacy cycle, the devices were visually verified to be clean on all surfaces, then tested for remaining soil as described above. The cleaning efficacy study was considered to have met the acceptance criteria if the remaining soil per total device surface area was measured to be less than 6.4 μg/cm^2^ for protein and less than 12.0 μg/cm^2^ for total organic carbon.

### 2.4 Steam sterilization and dry time validation for the magnetic bead insertion device

Steam sterilization and dry time were also validated by WuXi AppTec.

**FIGURE 2:**
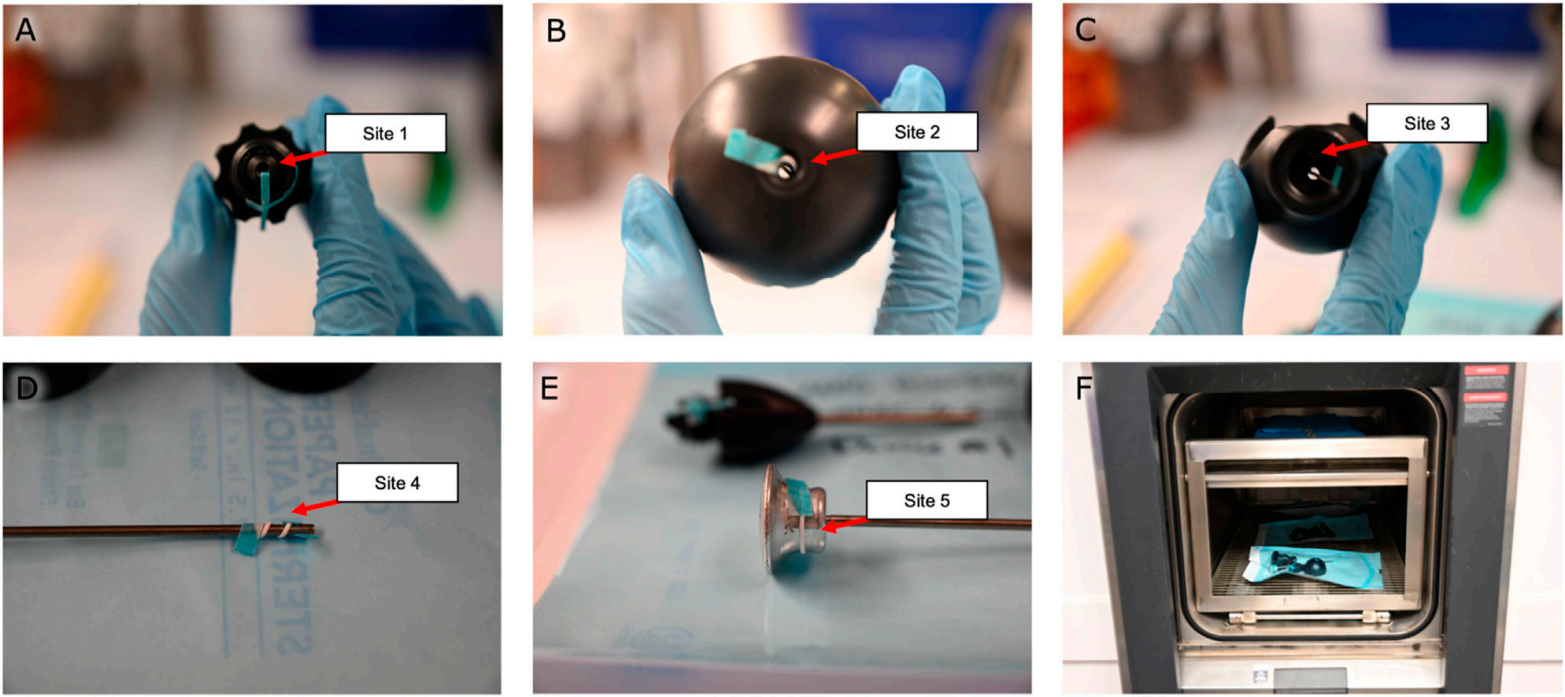
Steam sterilization study. Bioindicator strips were fed **(A)** into the titanium shaft, **(B)** into the cap, **(C)** through the center hole of the handle, and wrapped around the **(D)** distal and **(E)** proximal ends of the pushrod. **(F)** The disassembled devices were autoclaved for a half-cycle before being tested for sterility. Note the dunnage on the top rack used to simulate a full autoclave load.

For the validation of the process for sterilizing the magnetic bead insertion device, the three magnetic bead insertion devices were inoculated with five bioindicator strips each, with each strip containing 10^6^ *Geobacillus stearothermophilus* bioindicator spores (BI strip–Mesa Labs) having a decimal reduction value of 2 minutes at 121°C. As shown in Figures 2A–E, the bioindicator strips were fed into the titanium shaft, into the cap, through the center hole of the handle, and wrapped around the distal and proximal ends of the pushrod. The disassembled devices were then placed in autoclave pouches to be sterilized for a one-half cycle (1.5 min) at 134°C with dunnage overhead to simulate a full autoclave load. An additional bioindicator strip was handled similarly but was left outside the autoclave as a positive control. All bioindicator strips were retrieved and incubated for 7 days at 55°C–60°C while fully immersed in Soybean-Casein Digest Broth. The steam sterilization validation was considered to indicate a 10^6^ sterility assurance level (SAL) for a full 134°C autoclave sterilization cycle if the sterility test results for the test samples and positive controls were all found to be negative and positive, respectively. This steam sterilization process validation was conducted in compliance with ISO 17665-1: 2006, Sterilization of healthcare products–Moist heat–Part 1: Requirements for the development, validation and routine control of a sterilization process for medical devices.

For validation of the post-sterilization dry time of the magnetic bead insertion device, the three magnetic bead insertion devices were individually weighed separate from their autoclave packaging, packaged, then placed in an autoclave with dunnage overhead as was performed in the sterilization validation to simulate a full autoclave load (as shown in Figure 2F). The devices were autoclaved at 134°C for 3 minutes, then dried in the autoclave at a maintained temperature of 134°C for thirty minutes. Each device was unpackaged, and each device and packaging were inspected separately for residual moisture and weighed. This process was performed three times. The post-sterilization dry time study was considered to have met the acceptance criteria if no visible moisture was observed and neither the device mass nor the packaging mass was measured to have increased by more than 3%.

## 3 Results

### 3.1 Pyrogenicity testing

The pyrogenicity test resulted in a passing score, indicating that the magnetic bead implants of this study are non-pyrogenic. In the initial test, a temperature increase of 0.5°C was measured in one animal, suggesting continued testing. In the continued test, no additional temperature increases greater than 0.5°C were observed, and the summed temperature increase was 1.1°C (see Table 1).

**TABLE 1:** Pyrogenicity testing results. The following table lists the baseline temperatures for each rabbit in the 30 min before injection of the magnetic bead extraction, the maximum temperatures recorded for each rabbit 1–3 h post-injection, and the corresponding temperature increases (rounded up to zero), along with their individual pass/fail designations. The summed temperature increase across all eight rabbits is shown. No more than three animals received individual fail designations, and the summed temperature increase was less than 3.3°C, so the study result is non-pyrogenic.

	Rabbit	Baseline temperature (°C)	Maximum temperature (°C)	Temperature increase (°C)	Pass/Fail
Initial Test	1	39.2	39.2	0	Pass
	2	39.0	39.2	0.2	Pass
	3	39.0	39.5	0.5	Fail
Continued Test	4	39.4	39.5	0.1	Pass
	5	39.2	39.3	0.1	Pass
	6	39.2	39.2	0	Pass
	7	39.5	39.6	0.1	Pass
	8	39.4	39.5	0.1	Pass
	Summed Temperature Increase (°C) and Study Result			1.1	Pass (non-pyrogenic)

### 3.2 Chemical characterization and toxicological risk assessment

The chemical characterization detected, identified, and quantified 14 compounds and 7 elements (see Table 2). As listed in Table 2, all margins of safety for the identified compounds were greater than 10 and all margins of safety for identified elements were greater than 1, supporting a conclusion of tolerable risk of systemic toxicity, genotoxicity, carcinogenicity, and reproductive and developmental toxicity to patients.

**TABLE 2:** Chemical Characterization and Toxicological Risk Assessment Results. The table shows the chemicals found across all mass spectrometry analyses of all extractions of the magnetic beads, along with their CAS registry numbers, formulas, and structures. The rightmost column gives the margin of safety for each chemical, which is the ratio between the allowable limit and exposure level. All margins of safety are above 10 for compounds and above 1 for elements, supporting a conclusion of tolerable risk.

Chemical name	CAS	Formula	Structure	Margin of safety
(Z)-3-Nonen-1-ol	10340-23-5	C_9_H_18_O	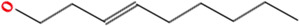	611
(Z)-docos-13-enamide	80399-99-1	C_22_H_43_NO	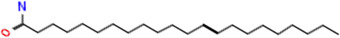	18,114
2,3-Dimyristoxypropyl tetradecanoate	115144-38-2	C_43_H_82_O_6_	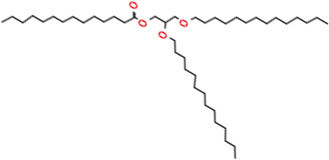	11
2-Ethyl-2-hydroxymethyl-1,3-propanediol, Trimethylolpropane	77-99-6	C₆H₁₄O₃	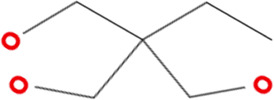	2,476
5,12-Dichlorotricyclo[8.2.2.24,7]hexadeca-1(12),4,6,10,13,15-hexaene	27414-57-9	C_16_H_14_Cl_2_	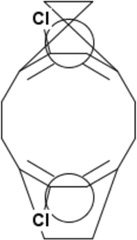	49
Hydrocarbon (C > 30)	NA	NA	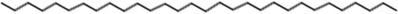	303
Hydrocarbon (C > 30)	NA	NA	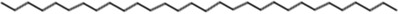	
Heptacosane	593-49-7	C_27_H_56_	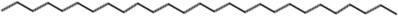	
Cyclic siloxane (D17)	150026-96-3	C₃₄H₁₀₂O₁₇Si₁₇	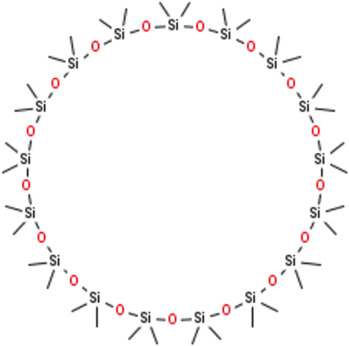	27
Diethylene glycol dibenzoate	120-55-8	C₁₈H₁₈O₅	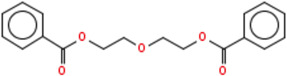	23,450
Methyl cinnamate	103-26-4	C₁₀H₁₀O₂	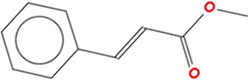	11,048
Triglyceride	NA	C_41_H_78_O_6_	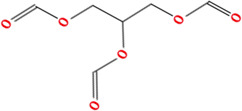	277,844
Irganox 1010 Isomer	NA	C_73_H_108_O_12_	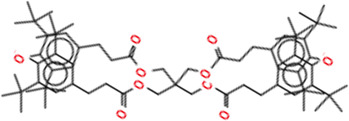	16,907
Acetone	67-64-1	C_3_H_6_O	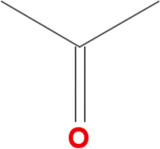	6,042
Sodium	7440-23-5	Na		15,315
Aluminum	7429-90-5	Al		4,833
Silicon	7440-21-3	Si		14,442
Calcium	7440-70-2	Ca		57,339
Zinc	7440-66-6	Zn		75,000
Nickel	7440-02-0	Ni		31
Copper	7440-50-8	Cu		938

### 3.3 Efficacy of cleaning the magnetic bead insertion device

The cleaning efficacy study met all acceptance criteria for the study (see Supplementary Table S1). Specifically, the soil per total device surface area was measured to be less than 6.4 μg/cm^2^ for protein and less than 12.0 μg/cm^2^ total organic carbon for all devices for both cleaning efficacy cycles, resulting in a passing score for the cleaning efficacy acceptance criteria and suggesting that the device can be safely cleaned in a healthcare setting according to the proposed cleaning process.

### 3.4 Steam sterilization and dry time validation for the magnetic bead insertion device

The steam sterilization validation met all acceptance criteria for the study, with all fifteen test samples testing negative and all three positive controls testing positive for the growth of *Geobacillus stearothermophilus*., indicating a 10^6^ sterility assurance level (SAL) for the magnetic bead insertion device after undergoing a full three-minute 134°C autoclave sterilization cycle.

The dry time validation met all acceptance criteria for the study. No moisture was observed on any of the devices during the dry time validation cycles. The mass of the devices and packaging decreased in all cases (see Supplementary Table S2), indicating that the magnetic bead insertion device can be completely dried using a 30-min autoclave dry time at 134°C following a full three-minute 134°C autoclave sterilization cycle.

## 4 Discussion

This work further solidifies the clinical biocompatibility of the magnetic bead implants and their insertion device, showing that the implants are non-pyrogenic and are considered to indicate low toxicological risk for each evaluated chemical for systemic toxicity, mutagenicity, carcinogenicity, and reproductive and developmental toxicity, in support of our first hypothesis. If the implants were considered pyrogenic, monitoring a patient’s temperature for a febrile response would be required. Instead, this determination of non-pyrogenicity following the medical standard for pyrogenicity testing of the implant provides confidence to a surgeon that the temperature of the patient does not need to be monitored due to the introduction of the implant. Additionally, the evaluation of low toxicological risk of the implant for systemic toxicity, mutagenicity, carcinogenicity, and reproductive and developmental toxicity is an important part of the determination that the implant is safe for use in humans.

The work also shows that the insertion device can be safely cleaned, sterilized, and dried in a healthcare setting, in support of our second hypothesis. This is an important conclusion for use of the insertion device as a reusable medical instrument.

### 4.1 Limitations

This work validated the non-pyrogenicity and biocompatibility of 3-mm-diameter spherical neodymium magnetic beads coated in 5 µm of gold and 21 µm of Parylene C and subjected to a stringent medical grade cleaning, packaging, and sterilization process. The results of this work may or may not be able to be extended to different magnet geometries, coating materials, and coating thicknesses. We do note that no gold was detected in the chemical characterization, and thus the gold coating may not be required, or it may be possible to have a thinner Parylene coating. However, the implant as designed offers extra confidence in its biocompatibility as a lifetime implant.

### 4.2 Conclusion

The results of this work, combined with the results of our previous work, provide confidence for the safe use of the magnetic implants in a forthcoming first-in-human clinical trial to evaluate magnetomicrometry as a permanent implant framework for bionic limb control.

## Data Availability

The original contributions presented in the study are included in the article and in the Supplementary Material, further inquiries can be directed to the corresponding author.

## References

[B1] Camp A. L. Astley H. C. Horner A. M. Roberts T. J. Brainerd E. L. (2016). Fluoromicrometry: a method for measuring muscle length dynamics with biplanar videofluoroscopy. J. Exp. Zoology Part A Ecol. Genet. Physiology 325 (7), 399–408. 10.1002/jez.2031 27488475

[B2] Griffiths R. I. (1987). Ultrasound transit time gives direct measurement of muscle fibre length *in vivo* . J. Neurosci. Methods 21 (2–4), 159–165. 10.1016/0165-0270(87)90113-0 3682872

[B3] Iacovacci V. Naselli I. Salgarella A. R. Clemente F. Ricotti L. Cipriani C. (2021). Stability and *in vivo* safety of gold, titanium nitride and Parylene C coatings on NdFeB magnets implanted in muscles towards a new generation of myokinetic prosthetic limbs. RSC Adv. 11 (12), 6766–6775. 10.1039/d0ra07989h 35423178 PMC8694929

[B4] Jordi M. A. Rowland K. Liu W. Cao X. Zong J. Yuan R. (2020). Reducing relative response factor variation using a multidetector approach for extractables and leachables (E&L) analysis to mitigate the need for uncertainty factors. J. Pharm. Biomed. Analysis 186 (July), 113334. 10.1016/j.jpba.2020.113334 32387747

[B5] Taylor C. R. Clark W. H. Clarrissimeaux, Seong Ho Yeon E. G. Carty M. J. Lipsitz S. R. Bronson R. T. (2022b). Clinical viability of magnetic bead implants in muscle. Front. Bioeng. Biotechnol. 10, 1010276. 10.3389/fbioe.2022.1010276 36394042 PMC9640959

[B6] Taylor C. R. Nott J. K. Nilmini H. R. Cohen J. M. Herr H. M. (2023). Non-pyrogenicity and biocompatibility of parylene-coated magnetic bead implants. bioRxiv. 10.1101/2023.09.01.555995 PMC10912624 38444650

[B7] Taylor C. R. Seong Ho Y. William H. C. Ellen G. C. Mary K. O. ’D. Roberts T. J. (2022a). Untethered muscle tracking using magnetomicrometry. Front. Bioeng. Biotechnol. 10, 1010275. 10.3389/fbioe.2022.1010275 36394028 PMC9640962

