# Supplementary material for: Non-pyrogenicity and biocompatibility of parylene-coated magnetic bead implants
Source: Front Bioeng Biotechnol. 2024 Feb 20;12:1290453. doi: 10.3389/fbioe.2024.1290453 (PMC10912624; doi:10.3389/fbioe.2024.1290453)
Supplement: Supplementary file 1 [file DataSheet1.PDF]

Supplementary Material

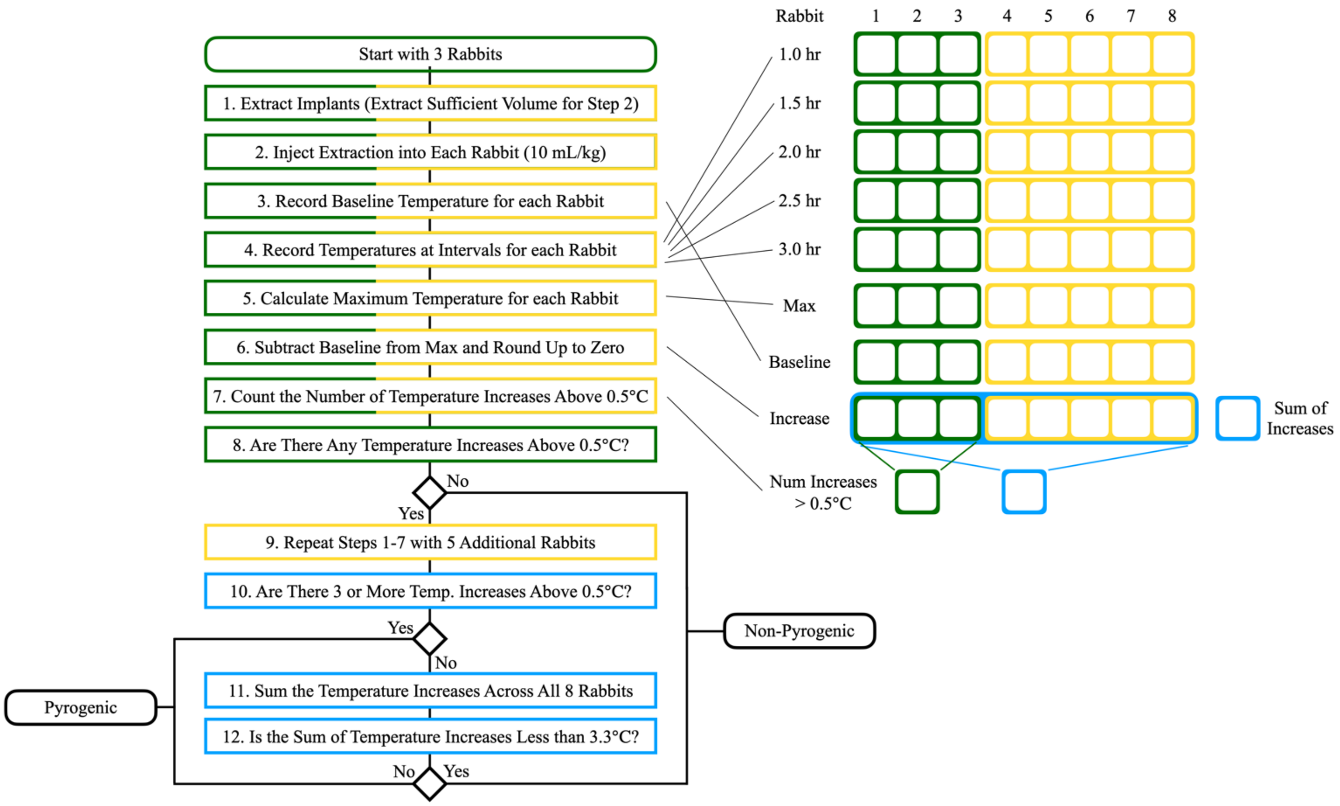

Supplementary Figure 1: Pyrogenicity Testing Process. *This flowchart describes the process we used to complete pyrogenicity testing. Note that steps 1-7 only need to be repeated if a temperature increase above 0.5°C is detected.*

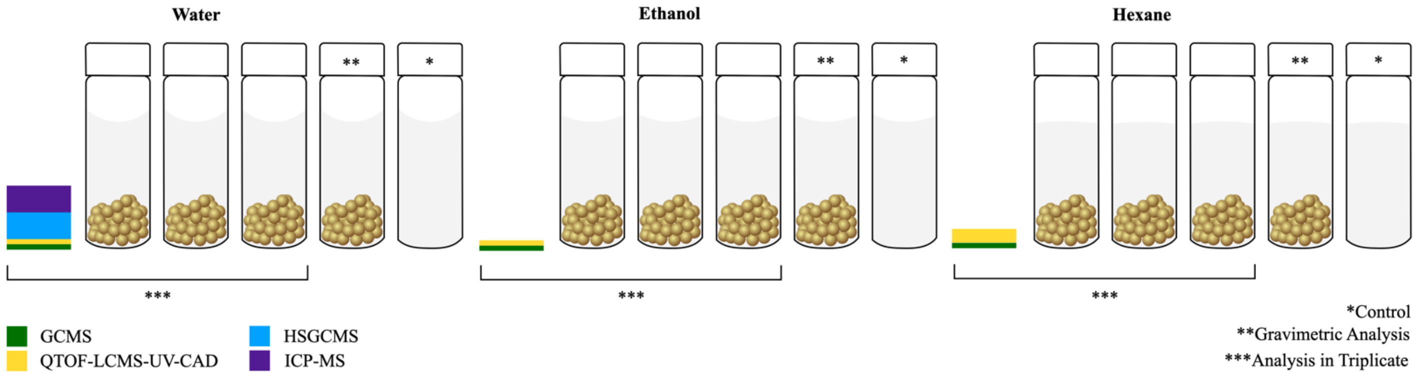

Supplementary Figure 2: Chemical Characterization Extractions. *This diagram shows the distribution of the magnetic bead extractions that were used to perform the chemical characterization.*

Supplementary Table 1: Cleaning Efficacy Results. *The soil per total device surface area was measured to be less than 6.4 µg/cm² for protein and less than 12.0 µg/cm² total organic carbon for all devices for both cleaning efficacy cycles, resulting in a passing score for the cleaning efficacy acceptance criteria.*

**Device**	**1**		**2**		**3**		
**Cleaning Efficacy Cycle**	**1**	**2**	**1**	**2**	**1**	**2**	
**Protein (µg/cm2)**	<0.6	<0.6	<0.6	<0.6	<0.6	<0.6	**Pass**
**Total Organic Carbon (µg/cm2)**	1.1	1.1	1.2	1.2	1.9	1.5	**Pass**

Supplementary Table 2: Dry Time Validation Results. *The pre-sterilization and post-dry-time weights are shown for each device across all three dry-time validation cycles, along with the percent weight increase from before sterilization to after drying. The weight decreased in all cases, resulting in a passing score for the dry time validation.*

	**Device**	**1**			**2**			**3**			
	**Dry Time Validation Cycle**	**1**	**2**	**3**	**1**	**2**	**3**	**1**	**2**	**3**	
**Device**	**Pre-Sterilization Weight (g)**	146.78	146.78	146.78	145.99	145.98	145.98	146.99	146.99	146.99	
	**Post-Dry-Time Weight (g)**	146.75	146.76	146.76	145.96	145.96	145.96	146.97	146.97	146.97	
	**Weight Increase (%)**	**-0.020%**	**-0.014%**	**-0.014%**	**-0.021%**	**-0.014%**	**-0.014%**	**-0.014%**	**-0.014%**	**-0.014%**	**Pass**
**Packaging**	**Pre-Sterilization Weight (g)**	7.60	7.53	7.55	7.56	7.65	7.50	7.54	7.61	7.55	
	**Post-Dry-Time Weight (g)**	7.40	7.33	7.35	7.38	7.46	7.29	7.38	7.44	7.35	
	**Weight Increase (%)**	**-2.644%**	**-2.591%**	**-2.687%**	**-2.303%**	**-2.470%**	**-2.695%**	**-2.070%**	**-2.260%**	**-2.571%**	**Pass**
